# Quantitative Representation of Mechanical Behavior of the Surface Hardening Layer in Shot-Peened Nickel-Based Superalloy

**DOI:** 10.3390/ma13061437

**Published:** 2020-03-21

**Authors:** Wu Zeng, Junjie Yang

**Affiliations:** 1 School of Aerospace Engineering, Tsinghua University, Beijing 100084, China; 2 Institute for Aero Engine, Tsinghua University, Beijing 100084, China

**Keywords:** surface hardened modified layers, pre-deformed material, instrumented indentation, microscopic zone mechanical behavior

## Abstract

Surface hardening treatment can usually introduce severe grain distortion with a large gradient in the surface layer. It results in mechanical properties being difficult to accurately determine through macroscopic tests due to the non-uniformity of the shot-peened material. In this study, the mechanical behavior of uniformly pre-deformed nickel-based superalloy IN718 was investigated with monotonic tensile tests and instrumented indentation tests. For the shot-peened material, the hardness distribution of the surface hardening layer after shot peening was identified through the instrumented indentation method. According to the stress–strain results of pre-deformed materials, Ramberg–Osgood model parameters could be presented with plastic deformation. Assuming the power-law relationship between hardness and plastic deformation, the plastic deformation distribution along the depth of the surface hardening layer was clarified. Based on the results, a method to identify the stress–strain relationships of hardened material at different depths was established. Finally, the finite-element simulations of the instrumented indentation test considered residual stress and strain hardening were built to verify the method presented herein. The results show that the solution to evaluate the mechanical properties of hardening layer materials in the microscopic zone is feasible, which can provide the foundation for the failure analysis of shot-peened materials.

## 1. Introduction

Surface hardening treatment such as shot peening treatment, is a crucial process to improve the fatigue life and reliability of key components such as discs in turbine engines. In particular, it is widely used in power machinery and aerospace industry [1,2,3]. Effects of surface hardening on surface material are mainly manifested in three aspects: residual stress, surface topography, and material distortion/microstructure changes. The treatment-affected subsurface layer becomes substantial for improving overall performance of the whole mechanical part [4,5,6]. It was found that surface hardening treatment introduces compressive residual stress on the surface of the material [7,8]. This compressive residual stress can significantly reduce the average stress of tensile loading of servicing components and therefore improve the fatigue life of the components. The literature [9,10,11] provides models for the effects of shot peening treatment on the fatigue strength and qualitatively describes the effect of the residual stress on the fatigue life. But there is no specific consideration or representation of the mechanical property at the surface layer. Klotz [12] used analytical analysis to predict the fatigue life of shot-peened IN718 specimens which well considered the residual stress and cold work of the surface hardening layer into the model. Generally, shot peening is considered to improve the fatigue property of mechanical components. However, previous studies have found that the low-cycle fatigue performance of shot-peened parts is significantly reduced [13,14,15,16]. The results indicate that it is not enough to qualitatively analyze the damage evolution of surface hardening from the modification of residual stress. Because the surface strengthening treatment of the material produces not only residual stress but also material distortion [17,18] and even phase transformation [19,20], we have to consider the effect of these factors on low-cycle fatigue failure.

Increasingly more research is concerned with the surface hardening layers of surface hardened materials. Kumar et al. [21] respectively reviewed the effect of individual microstructure feature, residual stress, and strain hardening on mechanical properties and fatigue crack mechanisms of nickel-based superalloys. The detailed and comprehensive understanding of surface hardened material would be useful for quantitatively clarifying the mechanical property. Koltz et al. [5] noted that shot peening introduced cold work at the surface and measured the microstructure, residual stress and cold work profile of the surface material. However, there are no quantitative descriptions of the mechanical behavior of the material. Zhang et al. [22] studied the gradient nanostructure surface layer of bearing steel after surface mechanical rolling treatment in terms of the hardness distribution and structure evolution. However, the mechanical properties distribution were not clarified. Zhao et al. [23] proposed that the surface-modified layer was inhomogeneous in the depth direction because of the gradient of strain and determined the mechanical properties of the surface-modified layer with expressions for the hardness and elastic modulus. Other studies [24,25] also measured the hardness distribution of the surface layers of surface hardened materials. Few studies have focused on the gradient distribution of mechanical properties, such as the stress–strain relationship, after mechanical surface hardening treatment. The mechanical properties of hardening layers are important for finite-element simulations of specimens because most of the present finite-element method (FEM) models use the uniform constitutive law for computation and neglect the gradient distribution of the properties.

The constitutive relationship of the surface hardening layer material is the basis for the discussion of the fatigue strength theory of surface hardened components. The FEM computation requires a plastic mechanics model of the material. In addition, the distortion of the hardening layer material affects the fatigue damage mechanism of the strengthened parts. Therefore, it is necessary to systematically study the mechanical properties of the hardening layer [5,26]. As for the characteristics of surface hardened material, it is difficult to study their mechanical behavior with conventional macro-scale methods. The microscopic zone mechanical behavior of the modified layer would be an interesting topic to discuss.

In the present work, the evolutions of the microstructure after large plastic deformation in a nickel-based superalloy IN718, including uniformly pre-deformed treated specimens and shot-peened specimens, were illustrated by electron backscatter diffraction(EBSD). Based on the analysis of grain distortion and mechanical behavior tests with uniformly pre-deformed specimens, a relationship between changes in hardness and changes in mechanical behavior was established, where the mechanical properties were obtained by monotonic tensile tests while the hardness was measured by instrumented indentation tests. According the results, a power-law relationship was proposed between hardness and plastic deformation. For the shot-peened specimens, the hardness distribution in the surface hardening layer was identified through the instrumented indentation method. Then, the macroscopic mechanical behavior distribution of hardened material at different depths was determined. Moreover, a finite-element simulation of the instrumented indentation test considered the residual stress and pre-strain hardening was used to verify the accuracy of the relations presented.

## 2. Material and Specimens

### 2.1. Material

IN718 nickel-based superalloy [27] is a kind of precipitationhardened nickel-based Ni-Cr-Fe alloy. In the present work, the investigated material is IN718 nickel-based superalloy, which is offered by Thyssen Krupp VDM GmbH in a solution-annealed state. The chemical composition of IN718 used is shown in Table 1.

The solution annealing was carried out according to the ASTM B 637-06 [28] solution treatment standard: 1000 °C for 1 h, followed by cooling in air, annealing at 720 °C for 8 h, and cooling down to 620 °C in the furnace, to be kept at 620 °C until the total heat treatment duration was 18 h.

### 2.2. Specimens

**Figure 1:**
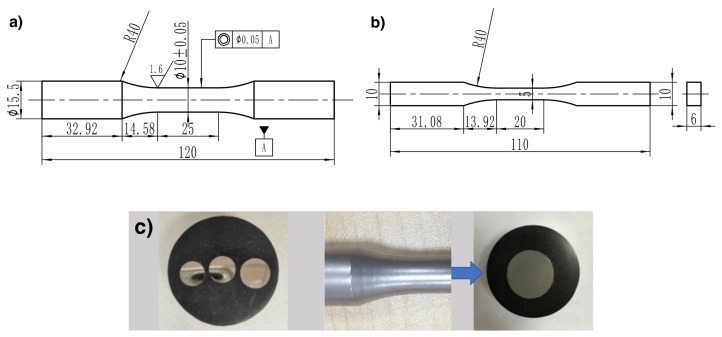
Specimen geometries tested in the present work. (**a**) The round specimens for instrumented indentation tests of the shot peening treatment. (**b**) The brick specimens for monotonic tensile tests of the pre-deformed treatment. (**c**) The samples for instrumented indentation tests of the pre-deformed and shot peening treatments.

The specimen geometries of the surface hardening treatment and pre-deformed treatment are shown in Figure 1. The round tensile specimens (Figure 1a were used to study mechanical properties with and without shot peening. The shot peening was performed with a compressed air machine at room temperature. The peening intensity was measured by Almen strips of type A. The two kinds of intensity were 0.1 mmA and 0.25 mmA, and the coverage was 100%.

The brick specimens in Figure 1b were fabricated to induce pre-deformations and to quantify effects of the material distortions on the mechanical property. The brick specimens were taken from the same batch of 16 mm round bar and pressed in the thickness direction with different compressive strains. The pre-deformations considered in the present work are −13.0% and −31.7%.

Figure 1c shows the specimens for instrumented indentation tests. For the shot-peened specimens, a cross-sectional sample is taken by a cutter and inlaid with Bakelite powder and polished to a mirror surface finish. Pre-deformed specimens of instrumented indentation tests were prepared in the form of smooth cylindrical specimens with a diameter of 6 mm and height of 5 mm and compressed with the MTS-Landmark hydraulic servo material test system. Three different pre-deformed samples were obtained by multiple sets of tests with uniform compression of −44.3%, −31.0%, and −18.7%.

### 2.3. Experiments

Microstructural characterization of base material and different treated material was conducted by a TESCAN MIRA-3LMH scanning electron microscope (TESCAN SEM) with EBSD. The pre-deformed samples were cut from the thickness direction. The cross section was scanned with EBSD and the direction was indicted in the figure. The deformation of the pre-deformed EBSD samples was 11.7% and 30.9%. The samples of shot-peened material for EBSD were prepared from the cross section of the round specimens. In order to clarify the difference of surface hardening layer, the scanning range covered the layer thickness in depth direction. Samples for EBSD analysis were subjected to a conventional metallographic procedure including grinding and electro-chemical polishing with 90% ethanol +10% perchloric acid at −25 °C. The EBSD data were processed using the HKL Channel 5 software package.

The instrumented indentation test was carried out on both the shot-peened specimens and the pre-deformed by using an Agilent G200 nanoindenter. For the shot-peened specimens, a cross-sectional sample was taken by a cutter and inlaid with Bakelite powder and polished to a mirror surface finish. Pre-deformed specimens of instrumented indentation tests were prepared in the form of smooth cylindrical specimens with a diameter of 6 mm and height of 5 mm and compressed with the MTS-Landmark hydraulic servo material test system. Three different pre-deformed samples were obtained by multiple sets of tests with uniform compression of −44.3%, −31.0%, and −18.7%.

The tests were carried out with displacement loading and the continuous stiffness standard method; the indentation depth was 2000 nm, the peak loading was maintained for 10s, and then the force loading was unloaded by 90%. To prevent interaction between the indentations, the interval distance was determined according to experience and the literature [29,30] showed that the interval between the pressure points was set to 3–5 times the diameter of the residual indentation, 50 μm. For the shot-peened material, the indentation points were arranged from the surface to the center in the diameter direction on the cross section of the sample; a 3 × 3 lattice was measured for the pre-deformed material, and the results were averaged to obtain the material test results.

## 3. Results and Discussions

### 3.1. Correlation between Crystallographic Microstructure and Macroscopic Property

#### 3.1.1. Microscopic Property of Pre-Deformed Material

**Figure 2:**
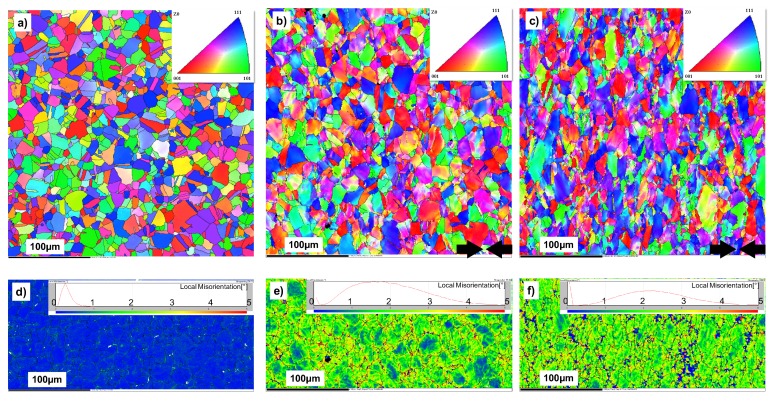
The microstructures of different treated IN718 samples were analyzed by EBSD: IPF maps (**a**–**c**) and KAM maps (**d**–**f**) of Base material (**a**,**d**), pre-deformation for 11.7% (**b**,**e**) and pre-deformation for 30.9% (**c**,**f**).

To illustrate the microstructures characterization of pre-deformed material, kernel average misorientation(KAM) maps were conducted. The inverse pole figures(IPF) maps with a large grain boundary show the thickness section of differently treated samples. Figure 2a,d show the microstructure of the base material without pre-deformation. It should be noted that the KAM maps (d, e, f) were calculated from IPF maps (a, b, c), respectively. This shows lower half of them to illustrate the local misorientation in the material. The IPF of the base material clearly shows the grain boundary and the equiaxed grains, with an average grain size of approximately 15–20 μm. Figure 2b,e show the microstructure of pre-deformed IN718 with 11.7% deformation, which is characterized by compressed grains without recrystallization. The arrows at the bottom of figure show the direction of pre-deformation treatment. Compared with those of the base material, the IPF maps show a little color change within the grains, revealing grain distortion because of pre-deformation. Figure 2c,f show the IPF maps and KAM maps for a compression of 30.9%. The maps show obvious grain distortion compared with those of the base material and 11.7% deformation. The microstructure is characterized by a smaller grain size and a larger local misorientation for the sample that is more severely deformed. The IPF maps of both 11.7% and 30.9% deformation show the same large grain boundary as the non-linear boundary as the base material. Because the sub-grain boundary increases with deformation, the maps show only the large grain boundary. The KAM maps of Figure 2d,f show the increase of local misorientation with the pre-deformation, which indicates the increase in dislocation density and its effect on the mechanical behavior of pre-deformed materials.

**Figure 3:**
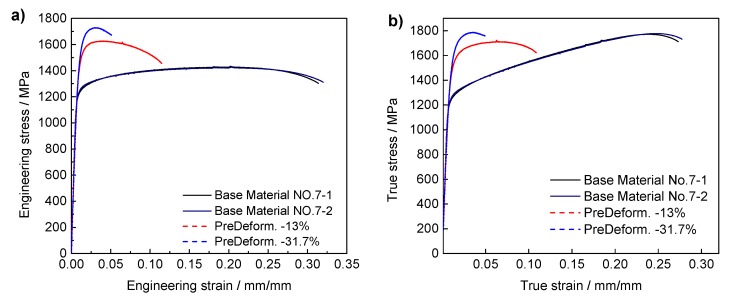
Results of the Stress–Strain relationship of the base material and pre-deformed IN718. (**a**) Engineering stress–strain curves. (**b**) True stress–strain curves.

The static material properties of the IN718 base material and pre-deformed specimens in Table 2 are summarized in Figure 3a. In Figure 3b, the true stress is plotted as a function of the true strain, which was calculated from the results measured by extensometer.

It can be seen that the yield and ultimate stresses of the pre-deformed material increase substantially with increasing pre-compressed deformation. Due to the pre-strain hardening effect of the pre-deformed specimens, the tensile test plastic hardening effect is significantly reduced compared to that of the base material and so as to the ratios of yield strength to ultimate strength. The fracture strain of the pre-deformed material is much lower than that of the base metal. This is due to the deformation of the grains under pre-plastic deformation conditions, resulting in a large number of dislocations and lattice distortions. The increase and movement of dislocations render the material deformation more difficult, i.e., the yield stress and tensile ultimate strength increase, and the plastic deformation ability of the material is reduced.

#### 3.1.2. Microstructure Characterization of Surface Layer after Shot Peening

**Figure 4:**
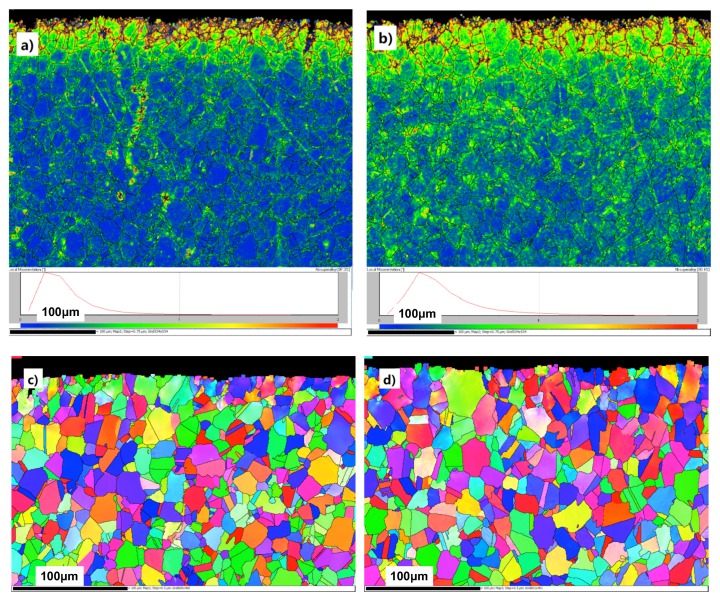
The microstructures of different shot-peened IN718 samples were analyzed by EBSD: (**a**) KAM map of 0.1 mmA, (**b**) KAM map of 0.2 mmA, (**c**) IPF map of 0.1 mmA and (**d**) IPF map of 0.2 mmA. The KAM maps illustrate the distribution of local misorientation in the material and the IPF maps illustrate the grain shape and size.

To characterize the grain morphologies of modified layer after shot peening, EBSD characterization was conducted, as shown in Figure 4. Compared to those of the base material shown in Figure 2a, the results of the EBSD tests on the surface area clearly show the changes in grain orientation, shape, and grain boundary orientation of the surface hardening layer of shot peening material with the increasing depth. The KAM maps clearly show the misorientation distribution of hardening layer of the shot-peened material. The local misorientation at the surface is much larger than that in the center material because of the impact of the high-speed shot peening. Also, the local misorientation and thickness of effect layer increase with the shot peening intensity. The literature reports that IN718 is a kind of austenite with a face-centered cubic structure [31,32,33] which has low stacking fault energy [31]. This would easily lead to twinning under the effect of shot peening.

#### 3.1.3. Stress–Strain Relationships of Materials with Severe Deformation

The stress–strain relationship of different pre-deformed IN718 can be described by the Ramberg–Osgood model [34], as follows:

(1) $$\varepsilon =\varepsilon_{e}+\varepsilon_{p}=\frac{\sigma }{E}+{\left(\frac{\sigma }{K}\right)}^{\frac{1}{n}}$$

where *K* is the strength coefficient shown the ability to resist plastic deformation. *n* is the exponent of the strain hardening effect of the material, reflected by the differential slope of the plastic strain and stress curve of the material [35,36].

**Figure 5:**
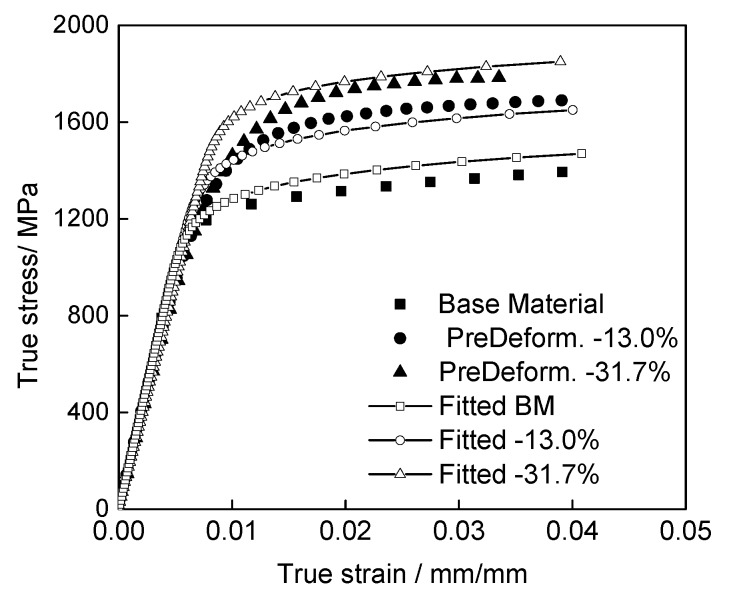
Results of the Stress–Strain relationship fitted with the Ramberg–Osgood Model of the base material and pre-deformed IN718.

It should be noted that there is a large difference between the fracture strains of the different pre-deformation specimens, as shown in Figure 3. Therefore, to ensure the comparability of the fitting results, the range of fitting stress–strain curves is selected to be 0–4%. The fitting results are shown in Figure 5. The points are the test results, and the dotted lines are the results of the fitting in the figure.

Since the mechanical behavior of the pre-deformed treated IN718 is affected by the pre-deformation, the relationship between the pre-plastic strain and the mechanical properties of the model is established according to the results of fitting the stress–strain relationship with the Ramberg–Osgood model. The relationships between the pre-plastic strain *D* and strength coefficient *K*/strain hardening exponent *n* should be established. According to the results, the relationship between the model parameters and the pre-plastic strain satisfies the following relationship:

(2) $$K=K_{0}+10.89D$$

(3) $$n=n_{0}-0.0566D$$

where $K_{0}=1832.49\;$MPa and $n_{0}=0.0628$ are the fitting parameters of the base material.

**Figure 6:**
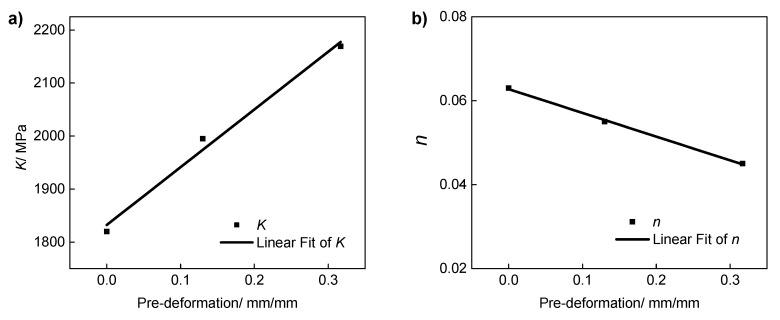
Variation in the mechanical properties with the deformation of pre-deformed IN718. (**a**) The fitted relationship between *K* and the pre-deformation. (**b**) The fitted relationship between *n* and the pre-deformation.

Figure 6 shows the fitted relationships between Ramberg–Osgood parameters and pre-deformation. Due to the effect of strain hardening, *K* increases with the increase of pre-deformation. In contrast, due to the hardening effects of the pre-plastic deformation, the hardening ability of the pre-deformed materials is reduced, i.e., *n* decreases with the increase of pre-deformation. According to the above results, we derive the stress–strain relationship with different pre-deformations.

### 3.2. Identification of Constitutive Behavior of Peened Materials

#### 3.2.1. Correlations between Micro Hardness with Microscopic Zone Mechanical Property

**Figure 7:**
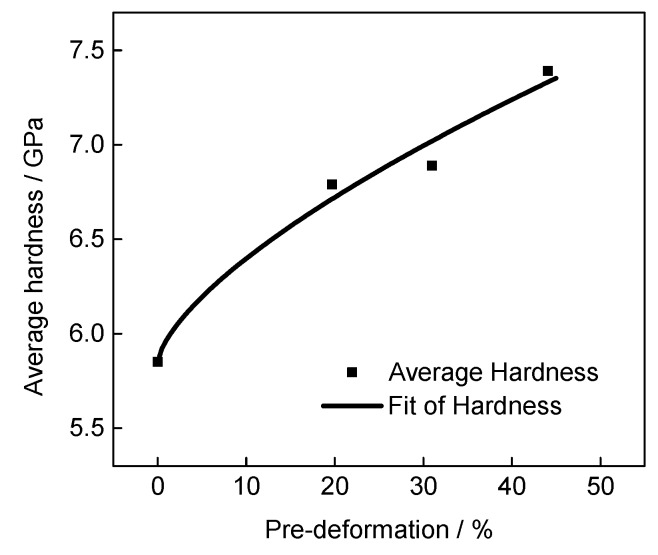
The fitted relationship between the hardness and pre-deformation of pre-deformed IN718.

The instrumented indentation hardness of the different pre-deformed materials was obtained through the instrumented indentation method, as shown in Figure 7, where the solid squares shown in indicate the average hardness. It is obvious that the hardness of the material increases monotonically with the increase of pre-deformation due to the strain hardening effect. According to the material plastic deformation dislocation theory, it can be qualitatively explained that the material dislocations proliferate with increasing plastic deformation. The growth in dislocation density hinders the deformation processes within the material, which increases the hardness of the material.

Hardness is a characterization of the comprehensive mechanical properties of materials. The mechanical properties of materials, including the plastic deformation and strain hardening ability, can be characterized by measuring the hardness [37,38]. Therefore, an empirical functional relationship between the hardness and plastic strain can be established for a specific material based on the test data. Furthermore, noted that the tendency of hardness increasing is approach to the power law, as shown in Figure 7. Based on this observation, the relationship between hardness and pre-deformation could be expressed with the following equation:

(4) $$H=H_{b}+\alpha D^{\beta }$$

where *D* is the plastic deformation; $H_{b}$ is the hardness of the base material, 5.85 GPa; α is the fitting parameter; and β is the impact of the strain hardening exponent on the hardness, reflecting the strain hardening ability of the material.

According to the pre-deformed specimens with instrumented indentation test results, the deformation and hardness of different pre-deformed materials can be fitted, and the fitted results are shown by line in Figure 7. The fitted α = 0.116, and β = 0.672. Establishing the relationship between the deformation and hardness provides a basis for identifying the plastic strain distribution of a microscopic zone of surface hardened material. Then, the microscopic zone mechanical property distribution of the modified layer of the surface hardened material can be identified.

The hardness of the modified layer of a surface hardened material varies over a gradient, and the hardness distribution can be measured with instrumented indentation tests. According the relationship between the hardness and the plastic strain shown in Equation (4), the equivalent plastic strain distribution of the modified layer can be obtained from the hardness distribution results.

**Figure 8:**
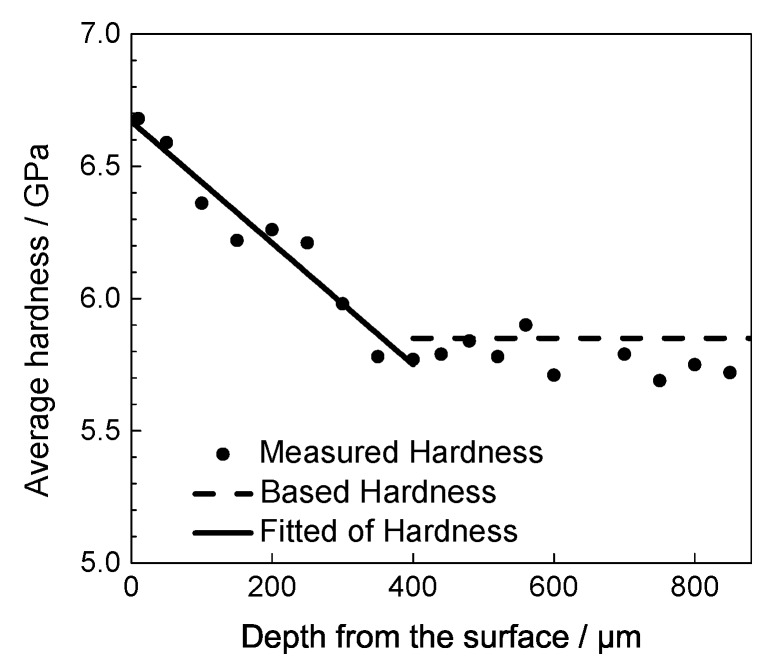
Hardness changes with depth for 0.1 mmA shot-peened IN718 specimens.

The material of the modified layer was tested with instrumented indentation according the diametrical direction. The hardness distribution test results are shown in Figure 8, where the round solid dots represent the experimental data. The horizontal axis represents the distance between the indentation position and the surface, which is the distribution along the depth. The results show that the hardness decreased with increasing depth, and the hardness trended to a stable value, i.e., the hardness property of the base material. The hardness decreased until a depth of 400 μm was reached, corresponding to the depth of the modified layer. According to the results, the hardness distribution is assumed to be a linear distribution, and the fitting result is shown by the solid line in the figure. The hardness of the base material was processed with averaging. The average result was 5.77 GPa, shown with dashed line. The linear distribution fitted relationship between the hardness and depth as follows:

(5) $$H=\left\{\begin{array}{cc} H_{s}-\frac{h}{h_{0}}\left(H_{s}-H_{0}\right), & h<h_{0}\\ H_{0}, & h\geq h_{0} \end{array}\right.$$

where $H_{s}$ is the hardness at the surface, $H_{0}$ is the hardness of base material, $h_{0}$ is the depth of shot peening hardness effected layer. $H_{s}$ for 0.1 mmA peening intensity is 6.67 GPa and $H_{0}$ is 5.77 GPa as showed in former section, determined based on the experimental results. *h* is the distance to the surface, μm.

#### 3.2.2. Mechanical Property of the Shot-Peened Subsurface Layer

According to the results before, the hardness of the pre-deformed material and the shot-peened material can be evaluated by the instrumented indentation tests. The relationship between hardness and plastic deformation can be established, i.e., according to Equations (4) and (5), the distribution of plastic deformation of the shot-peening-modified layer material along the depth direction can be obtained. The relationship is as follows:

(6) $$D={\left[\frac{(H_{s}-H_{b})-0.0023h}{\alpha }\right]}^{1/\beta }\;h\in \left(0,400\right)$$

**Figure 9:**
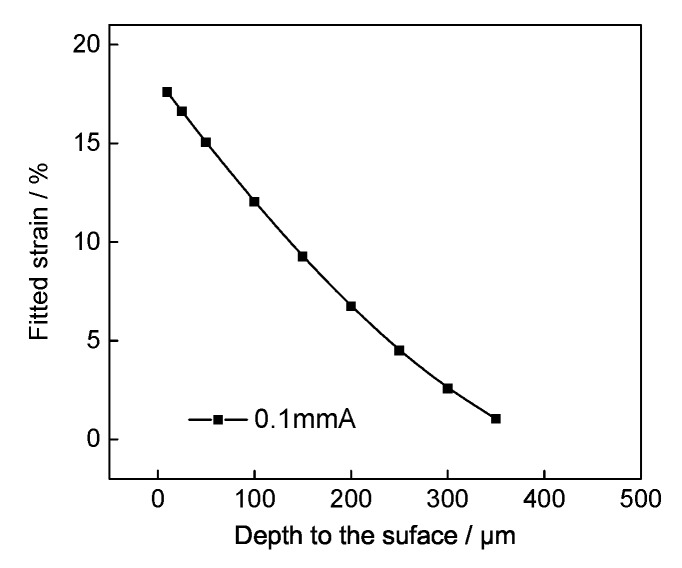
Fitted strain changes with depth for 0.1 mmA shot-peened IN718 specimens.

Please note that the affected thickness of shot peening was 400 μm for the specimens treated with 0.1 mmA, as shown in Figure 8. Therefore, the plastic deformations, *D*, can be obtained at the different depth from the surface. Figure 9 shows the fitted results, it can be seen clearly that the plastic strain decrease with increasing depth, and the calculated maximum plastic deformation *D* was 17.6% at the surface.

**Figure 10:**
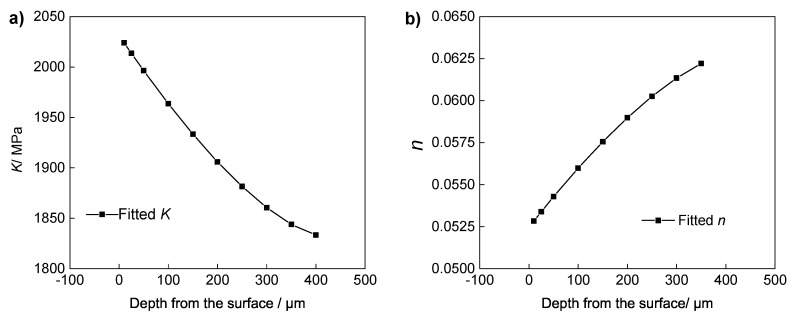
Parameters *K* and *n* changing with depth for 0.1 mmA shot-peened IN718 specimens. (**a**) The fitted distribution of *K* with depth. (**b**) The fitted distribution of *n* with depth.

Based on the relationship of the plastic strain *D* with the parameters *n* and *K* shown in Equations (2) and (3), we can derive the variation of *n* and *K* with increasing depth relative to the surface. The stress–strain relationship at different depths of the shot-peened modified layer materials can be obtained. The parameters *K* and *n* of the Ramberg–Osgood relationship with increasing depth are shown in Figure 10.

**Figure 11:**
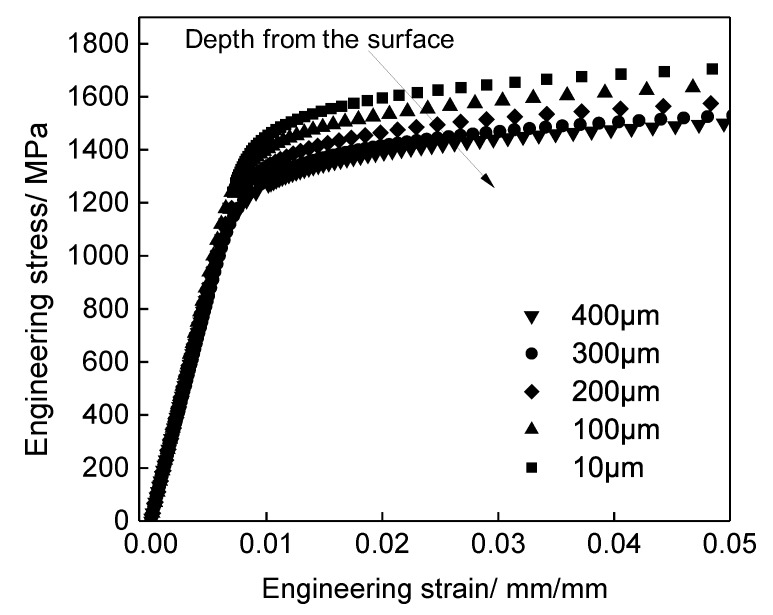
The derived material stress–strain relationship at different depth.

According to the above experimental results and analysis and fitting results, the stress and strain curves of the modified layer material at different depths could be obtained, shown in Figure 11. The calculation results showed that the material behavior of stress decreased with increasing depth. Additionally, it can be seen that the changing rate of stress and strain at the near surface layer was much larger. The characterization of the stress–strain relationships of materials at different depths provides a basis for constructing the finite-element simulation model of a material layer modified by shot peening.

#### 3.2.3. FEM Simulation of the Instrumented Indentation Tests

The present work, based on instrumented indentation tests and monotonic tensile tests combined with pre-deformation treatment, established the plastic strain-hardness relationship and the hardness–stress–strain relationship. This relationship was based on the phenomenological test induction and model hypothesis. As a result of the summary, no theoretical derivation has yet been established. Moreover, the influence of residual stress was not eliminated during the instrumented indentation experiment. Therefore, the material mechanical properties of the modified layer could be verified by FEM simulation.

**Figure 12:**
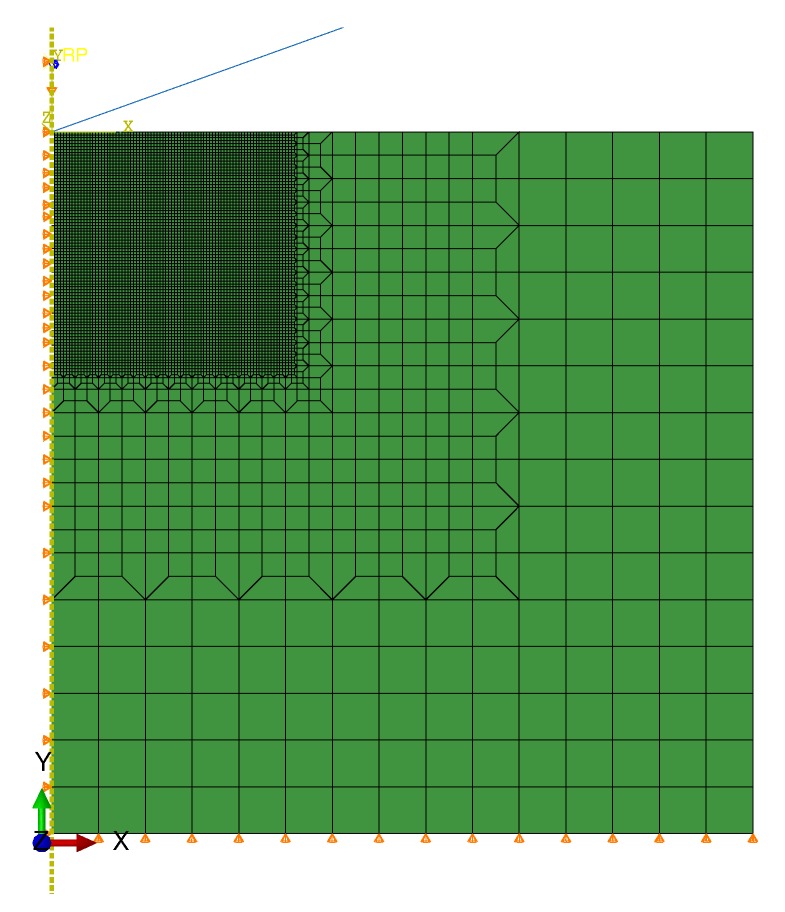
Mesh and Boundary Condition of Instrumented Indentation Simulation Model.

The boundary of the FEM simulation model for instrumented indentation tests was simplified without affecting the discussion of the computation results. A simplified two-dimensional axisymmetric model [39] was used in this simulation. For the limit of the two-dimensional axisymmetric model and the tetrahedral structure of the Berkovich indenter, an equivalent conical indenter with the same contact area function A(h) was created. The equivalent cone angle of the cone was 140.6${}^{\circ }$. The indenter in the model was simplified as a rigid diagonal line with an angle of 70.3${}^{\circ }$ from the center axis. The simulation was set to neglect the friction between the indenter and the specimens. The two-dimensional model was set at 30 × 30 μm. The mesh of the indenter contact area of the indenter was refined, and the grid was gradually thickened away from the indentation area to improve the computation efficiency, shown in Figure 12.

The meshes adopted a solid axisymmetric four-node linear reduction integral element(CAXR4), and the total number of elements was 7533. The material properties were obtained by the above-mentioned study to obtain the stress–strain curves and Young’s modulus measured by instrumented indentation tests of the modified layer materials. It should be noted that the influence of residual stress was considered in the simulation model. The residual stress of shot-peened specimens along depth of the modified layer could be measured with X-ray diffraction(XRD) tests. Actually, the distribution is continuous and different at different depth. But the residual stress of SEM simulation model was supposed to be uniform in the certain area. The residual stress was defined as an initial boundary condition with “Predefined Field” of the finite-element ABAQUS software.

**Figure 13:**
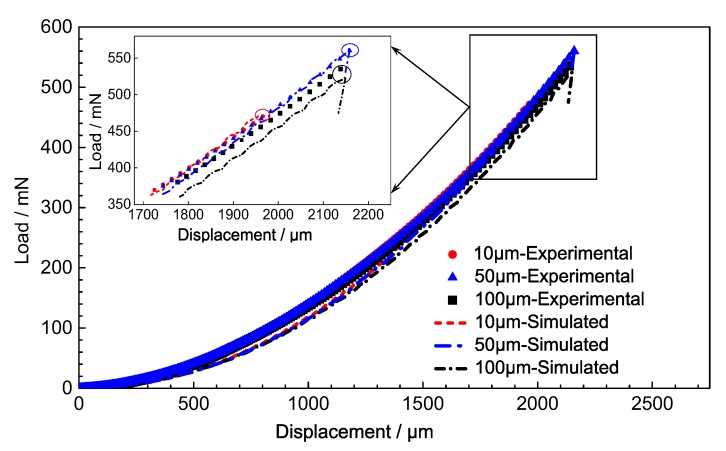
Comparison between experiments and simulation of instrumented indentation in the 0.1 mmA shot-peened IN718 material.

That is, the instrumented indentation loading test process was simulated with FEM. Three points located at different distance from surface of shot peening specimens, 10, 50 and 100 μm, were calculated. The load-displacement relationship of different points at different depth was obtained, and the results are shown in Figure 13. The solid points of different shapes in the figure represent the load-displacement test data at different depths of the test position on the surface of the test piece, and the corresponding dash or dash dot lines indicate the result of the FEM simulation results.

Three points in the calculation of the instrumented indentation test simulation could be used to calculate the maximum load $P_{max}$ of the instrumented indentation test accurately. Additionally, the load-displacement curves fit very well. The results verified the micro zone stress–strain relationship of the shot-peening-modified material.

The results show that the material plastic deformation and material hardening effect was reduced with pre-deformation. As for the effect of pile up during the instrumented indentation test, the actual contact area of indentation was larger than the ideal theoretical contact area, so the results of experimental curves were slightly higher than the FEM-calculated results without considering the effect of pile up. The contrast results are shown in Figure 13.

## 4. Conclusions

Shot peening treatment usually introduces a large gradient in mechanical behavior in the surface hardening layer in the material. The mechanical properties are difficult to determine through the general macro test methods. In the present study, combined the macro monotonic tensile test with the micro hardness measurement, the distribution of stress–strain relationship in the hardening layer was quantitatively characterized with pre-deformation specimens and shot-peened specimens. Some novel conclusions could be drawn as follows:

1. Both pre-deformation treatment and shot peening can lead to severe distortion in materials. Due to the increase of dislocation density, hardness and the strength coefficient can significantly increase with pre-deformation and shot peening intensity, while the fracture strain and the strain hardening exponent of the material can be decreased. A relationship between parameters of the Ramberg–Osgood model and hardness was established based on the test data of pre-deformation specimens. The relationship is adapted to severe distortion materials including shot-peened materials.
2. There are severe plastic deformation and complicated hardness distribution in the surface hardening layer for the shot-peened material. In the paper, hardness was presented to characterize the mechanical properties of materials, including the plastic deformation and strain hardening effect. Based on the hardness distribution in the surface hardening layer for the shot-peened material identified through the instrumented indentation method, the plastic deformation distribution in the surface hardening layer was derived. Furthermore, the stress–strain curves at different depths of the surface hardening layer was obtained according to the relationship between parameters of the Ramberg–Osgood model and hardness.
3. Based on the distribution of mechanical behavior, the finite-element model of the instrumented indentation test was established considered the residual stress and pre-strain hardening. The load-displacement curves corresponding to the instrumented indentation test results were obtained and agreed well with the experimental results. It indicates that the present work can offer a solution to evaluate the mechanical properties of surface hardening layer in the microscopic zone, providing the foundation for the analysis of fatigue failure of surface hardened materials.

## Figures and Tables

**Table 1:** Nominal chemical composite of IN718 (wt, %).

Cr	Ni	Fe	Mo	Nb	Ti	Al	C	Si	Co	Cu
18.51	53.31	17.95	3.05	5.30	0.98	0.54	0.03	0.05	0.11	0.04

**Table 2:** Static mechanical property (Engineering Stress–Strain) of the IN718 base material and pre-deformed material.

Specimens	Elastic Modulus	Yield Stress	Ultimate Stress	Fracture Strain
	(GPa)	(MPa)	(MPa)	(%)
Base Material	206.9	1197.0	1440.0	31.63
−11.7%	197.1	1343.0	1625.1	11.46
−30.9%	188.6	1401.8	1728.5	5.08
